# A proposed framework for the interpretation of biomonitoring data

**DOI:** 10.1186/1476-069X-7-S1-S12

**Published:** 2008-06-05

**Authors:** Peter J Boogaard, Chris D Money

**Affiliations:** 1 Shell Health, Shell International bv, PO Box 162, 2501 AN The Hague, Netherlands; 2 Exxon Mobil Petroleum & Chemical, Hermeslaan 2, B-1831 Machelen, Belgium

## Abstract

Biomonitoring, the determination of chemical substances in human body fluids or tissues, is more and more frequently applied. At the same time detection limits are decreasing steadily. As a consequence, many data with potential relevance for public health are generated although they need not necessarily allow interpretation in term of health relevance. The European Centre of Ecotoxicology and Toxicology of Chemicals (ECETOC) formed a dedicated task force to build a framework for the interpretation of biomonitoring data. The framework that was developed evaluates biomonitoring data based on their analytical integrity, their ability to describe dose (toxicokinetics), their ability to relate to effects, and an overall evaluation and weight of evidence analysis. This framework was subsequently evaluated with a number of case studies and was shown to provide a rational basis to advance discussions on human biomonitoring allowing better use and application of this type of data in human health risk assessment.

## Introduction

Biomonitoring, the measurement of the concentrations of chemical substances in human body fluids and tissues, has been routinely applied in industry and parts of the public health arena for more than 50 years [1]. The continuously increasing availability of analytical methodologies in combination with a constant decrease in detection limits has rendered biomonitoring both more accessible and more sensitive. As a consequence, biomonitoring is more and more frequently applied in various health settings. This leads primarily to an increase in the available knowledge on the extent of human exposure to chemical substances. In addition, it may create a number of opportunities for improving human health risk assessment because it triggers new research investigating the links between low-level exposures, adverse health effects, and potentially vulnerable population groups. On the other hand, the use of biomonitoring data creates a number of challenges, not least because the nature of biomonitoring findings is often heterogeneous. As a consequence, there is an emerging need to ensure that biomonitoring data are interpreted within the boundary in which they can be reliably applied. This is of particular interest in view of the increased attention for personalised exposure information from the general public when adverse health effects are merely suspected from environmental exposure to chemicals [2]. It also stresses the importance of scientifically sound and reliable interpretation of biomonitoring data. In this respect, it should be emphasised that much apposite learning can be gleaned from examining the accumulated experiences arising from the past use of biomonitoring in occupational and public health settings.

ECETOC is a non-profit, non-advocacy, scientific organisation funded by over 40 of the major chemical producing and using companies in Europe. It was founded in 1978 with the aim of improving the understanding of the human and environmental risks arising from the manufacture and use of chemicals. ECETOC has published over 300 peer reviewed reports and publications, and holds the status of a non-governmental organisation (NGO) status at the WHO and at several other world and European bodies.

Given this emerging trend for increased availability of biomonitoring data, ECETOC constituted a Task Force, comprising members from industry, academia and non-governmental organisations, to develop a framework in which biomonitoring data can be consistently evaluated and which can be used to foster a consistent basis for the application of biomonitoring data for risk assessment purposes.

The Task Force was made up of the following persons:

• Dr. P.J. Boogaard (chairman), Shell International, NL;

• Prof. Dr. P.B. Farmer, Univ. Leicester, UK;

• Dr. M. Holt, ECETOC, B;

• Prof. Dr. L.E. Knudsen, Univ. Copenhagen, DK;

• Dr. L. Onyon, WHO, CH;

• Dr. S.H. Robison, Procter and Gamble, USA;

• Prof. Dr. G. Schoeters, VITO, B;

• Dr. G.D. Stropp, Bayer HealthCare, D;

• Dr. M.F. Wilks, Syngenta Crop Protection, CH;

• Dr. W. Will, BASF, D.

This article will give an overview of the framework as laid out by this Task Force and identify a number of areas that require further development and discussion to ensure the reliable and responsible use of human biomonitoring data.

## The need for human biomonitoring data in risk assessment

Biomonitoring is a general term that includes various levels of determining exposure and effects and comprises the following subcategories:

1. Biological monitoring or biomarkers of exposure (also: internal dose or body burden);

2. Biochemical effect monitoring or biomarkers of effective dose (also: tissue dose);

3. a. Biological effect monitoring or biomarkers of effect

b. Clinical parameters or biomarkers of disease.

**Figure 1:**
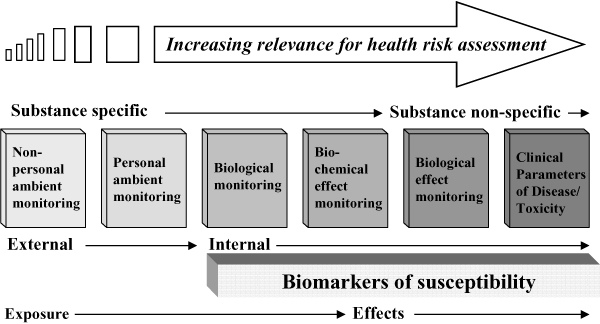
Monitoring techniques as part of the exposure-disease continuum.

In addition, both phenotype and genotype may be referred to as biomarkers of susceptibility. Biomonitoring is part of the suite of monitoring techniques in the exposure-disease continuum as illustrated in Figure 1. In developing the framework for the interpretation of biomonitoring data, the Task Force focussed on biological monitoring and biochemical effect monitoring as these both provide chemical-specific internal exposure data whereas the data in the third category (biomarkers of effect and of disease) can not directly be linked to chemical exposure. This does not imply that that the Task Force considered these data not useful. On the contrary, like the other biomonitoring data they form part of the large body of human experience data, which various authorities have recognised, needs to be incorporated into the risk assessment process to improve the utility and robustness of the assessments [3-5].

Human data can present in many forms, ranging from the findings of epidemiological studies to the results of workplace or public health surveillance programmes [6]. Amongst various attributes, human experience data in its various forms enables;

1) New health effects to be identified e.g. through epidemiology or case studies;

2) Calibration of the results from animal findings against the human experience;

3) Human effect levels to be defined with more certainty.

For risk assessment it is crucial that a reliable causal relationship can be established between a specific human health effect and a specific (chemical) exposure. Human biomonitoring, especially at the public health level, can play a crucial role in establishing such a causal relationship. Therefore, at the European level, the incorporation of human data into the risk assessment process was specifically encouraged [4]. Nevertheless, this focus seems to fade in the new chemicals legislation (REACH) that states that human test data are 'generally not acceptable'. However, this statement may be a mere reflection of some EU member states' view not to encourage human volunteer testing. If not, it would seriously undermine the very aims of REACH since it would not only deny the intrinsic value that human data itself brings to the risk assessment process but also fail to recognise that many health effects of concern cannot be identified from animal studies e.g. respiratory sensitization, myeloid leukaemia, headaches, etc. The use of human data is further reinforced by animal welfare considerations since the need to initiate animal testing is questionable if good quality data from human experiences are available. In addition, the use of human data reduces uncertainty since assessment factors for interspecies extrapolation are superfluous when human data are used.

### Guidelines for the interpretation of human biomonitoring data

**Figure 2:**
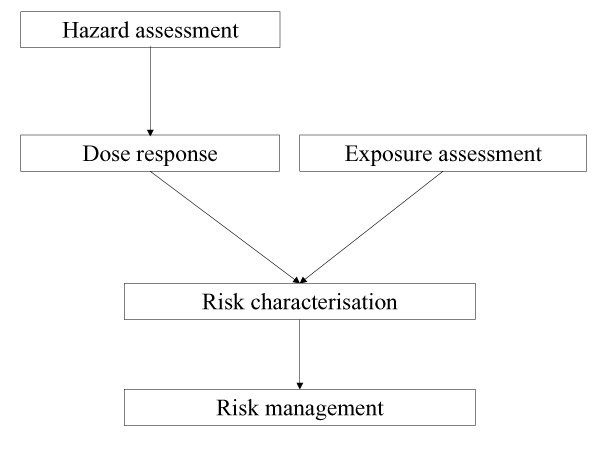
Process for risk characterisation and management (adapted from [4]).

The Task Force accepted the risk management paradigm as formulated by the European Union (Figure 2), which requires a dose-response relationship together with exposure data to characterize the health risk of a specific chemical hazard to subsequently decide whether the risk is such that management is required. In the standard European Union risk assessment process, the risks for industrial operators, for consumers and for 'man through the environment' is assessed. The last category relates to health risks of the general public potentially caused by exposure from chemical substances in the environment. Human biomonitoring is ideally suited to assess this level of exposure as, by its very nature, it integrates all routes of exposure (oral, dermal and by inhalation) and provides currently the most reliable and relevant integrated exposure metric.

The Task Force identified four specific elements which, dependent on the degree to which each is supported, allow any set of biomonitoring data to be evaluated with respect to the extent to which it can be applied to different stages of the risk assessment process. The four elements are:

#### • Analytical integrity

The analytical integrity includes (1) consideration of internal quality control factors, such as the availability of standard operating procedures for the pre-analytical phase (sample collection, sample storage), the analytical phase (actual analytical procedures, quality controls including external quality control when available), as well as the post-analytical phase (statistical analyses of the data, reporting of the results), (2) standardisation of conditions for sample collection and storage, (3) determination of recoveries, reproducibility and accuracy (which should all be in an acceptable range) for analytical methods used, (4) availability of reference standards, and (5) the use of control samples and 'blank' samples, which should ensure that there is no contamination or artifactual production of the analyte caused by the sample isolation or analysis. A typical example of this can be seen with oxidative DNA damage: for the same biological sample different methodologies resulted in measured concentrations of 8-oxo-7,8-dihydroguanine (the most ubiquitous oxidation product of guanine) that differed more than 2 orders of magnitude. [7]. The reasons for this discrepancy was found to originate from adventitious oxidation of guanine during the isolation of the DNA and subsequent derivatisation.

#### • Ability to describe dose (toxicokinetics)

For risk assessment of a specific chemical substance knowledge of the dose, defined as the exposure to this substance as a function of time, is essential. Biological monitoring and biochemical effect monitoring provide a snapshot of those substances or their metabolites present in the body independent of the route of exposure and thus, make it possible to draw conclusions on the integrated dose. For differentiating between exposures, the monitoring method must be selective and measure exclusively the object of the biomonitoring programme. This selectivity depends not only on analytical specificity, but also, especially in situations in which the biomarker is not the parent chemical, on other sources of the metabolite from other chemicals. Not only is the choice of a suitable biomarker essential, but its disposition kinetics are also important with respect to the possible biological variability relative to external chemical exposure. Furthermore, to be able to relate biomonitoring data back to exposure, a detailed knowledge of human metabolism and toxicokinetics, including their biological variability [8], is desirable, if not, essential.

#### • Ability to relate data to effects

Important aspects for the ability to relate biomarker concentrations to effects are (1) the knowledge of background levels in the general population, (2) the relation between external and internal exposure, (3) the relation between biomarker concentration and the total dose, (4) the estimation of the inter- and intra-individual variability, and (5) the evaluation of confounding factors (including systematic errors) that can affect the biomarker. The Task Force recognised that there is a considerable lack of knowledge of low-dose response relationships which makes it difficult to establish causal relationships between biomonitoring (or other exposure) data and observed effects. Criteria classically used to establish such a causality may be helpful here, such as those proposed by Bradford-Hill [9] and further developed by Vineis and Porta [10], whilst the overall validity needs to be considered by evaluating analytical integrity, the relevance, specificity and sensitivity. This may, for example, be done by applying the framework developed by IPCS, which evaluates the collective information from diverse datasets in a structured manner to provide objective assessments of the state-of-the-science of determining causality between exposure to a chemical and selected health outcomes [11].

Moreover, any biomarker study related to health effects should be hypothesis driven [12]. The hypothesis of a causal relationship between biomarkers of exposure and observed effects needs to be set in advance, prior to the initiation of the study. The hypothesis under study should be based on an understanding of the mode of action of a chemical wherever possible, on animal data, or on comparative epidemiological studies. A good example of this is given by the studies on aflatoxin B1 biomarkers, where the relationship between aflatoxin ingestion and hepatocellular carcinoma was evident from epidemiological studies. In a series of elegant studies, it was shown that there was no statistically significant association between the risk of liver cancer and dietary aflatoxin intake (i.e. external exposure) but a highly significant correlation between hepatocellular carcinomas and the internal exposure to aflatoxin, as measured by the determination of urinary aflatoxin B1 metabolites, especially its major DNA adduct (aflatoxin-*N*7-guanine) [13-16]. Moreover, the effectiveness of intervention policies could be followed through biomonitoring. Dietary chlorophyllin reduced aflatoxin B1 DNA adducts in rats and prevented against hepatocarcinogenesis and a significant reduction in urinary aflatoxin-*N7*-guanine was also measured in human populations treated with this chemoprotective agent [17]. Similarly, dramatic reductions in blood lead concentrations in children were observed following the introduction of policies to ban lead in paints and automotive fuels [18-20].

#### • Overall evaluation and weight of evidence

Evaluation of causal criteria that link an exposure to a specific effect can be highly complex. It often involves integration of data from many studies that differ in terms of experimental conditions and the parameters examined. This is termed the 'weight of evidence' approach and used in different areas of evaluation (see for instance [21]). The weight of evidence approach is the evaluation of all available data on a specific compound or hypothesis and has to be done as a case-by-case evaluation usually requiring expert judgment.

**Figure 3:**
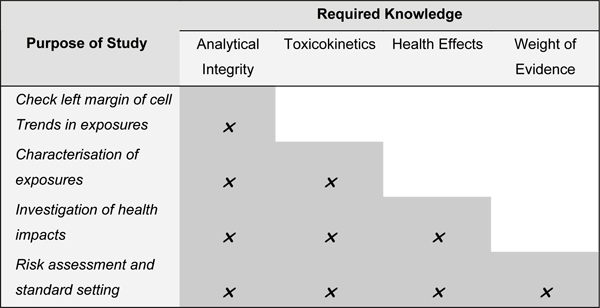
The Proposed Framework for the Evaluation of Biomonitoring Data.

The approach followed by the Task Force to categorise data builds off the general guidance contained in earlier publications [22] and is shown in Figure 3. Analysis of the extent to which any set of biomonitoring data are supported by the 'required knowledge', enables to determine the main risk assessment functions that these data can be reliably be applied to. Specifically, these functions are to:

• Establish exposure patterns and trends;

• Characterise the nature of exposures;

• Investigate linkages between exposure and adverse health effects; and

• Facilitate risk management, including standard setting.

### Recommendations and conclusions

The proposed approach to interpreting biomonitoring results is intended both to offer a considered view of the available science aànd to serve as a catalyst for stimulating discussion on some of the broader issues presented by the application of biomonitoring technologies today [23]. The utility of the approach was tested in a series of illustrative case studies, which made it possible to identify where different types of biomonitoring data can be reliably used and to indicate their relative importance. It also allowed identifying where and how they can be incorporated into a framework that provides the basis for their consistent interpretation whilst accounting for the current level of understanding of the supporting science.

In developing the framework, a number of areas were identified as requiring further work. These included (1) further validation to better establish the boundaries within which biomonitoring can be reliably applied – specifically, further work should be undertaken to more clearly define how biomarkers of effect and susceptibility (see Fig. 1) could reliably be incorporated, (2) development of a more extensive library of case studies that would serve as a training tool to help risk assessors etc. understand how different forms of biomonitoring data should be evaluated and applied, (3) development of guidance on how study findings should be communicated to different interest groups; this guidance would need to cover the communication of results to individuals and groups, as well as including the wider communication of findings to external audiences, and (4) clarification on the rules and considerations that govern the ethics of how biomonitoring surveys and programmes are initiated, managed and maintained; rules which could provide a basis for such guidance exist, but the extent to which biomonitoring surveys, particularly those undertaken in the public health setting, address these issues is currently inconsistent and the development of clear and concise ethically-based guidance in this area would help minimise this. In addition, clear and succinct guidance on the practical aspects of how and in what context such studies might be undertaken in practice would be helpful. A number of these areas are currently being addressed by ESBIO (the Expert team to Support BIOmonitoring in Europe, is an expert network is funded by the European Commission (Directorate-General Research) under the 6^th ^Framework Programme for Research and Technological Development in close cooperation with Directorate-General Environment .

In conclusion, a framework was developed that provides a rational basis on which to further the discussion on human biomonitoring and will hopefully lead to the better use and application of this type of data in human health risk assessment. The full report can be downloaded from the ECETOC website at .

## Competing interests

The authors declare that they have no competing interests.
